# Dynamics and Dependencies in Regional Collaboration for Biodiversity Restoration: Reflections from the Netherlands

**DOI:** 10.1007/s00267-024-01958-6

**Published:** 2024-03-07

**Authors:** Sabine Baumgarten, Noelle Aarts, Jan M. Fliervoet, Lotte Krabbenborg

**Affiliations:** 1 Radboud University, Institute for Science in Society, Nijmegen, The Netherlands; 2 Research group Communication, Participation & Social-Ecological Learning, Van Hall Larenstein University of Applied Sciences, Velp, The Netherlands

**Keywords:** Biodiversity restoration, Social-ecological systems, Multi-stakeholder collaboration, Collaborative governance, Self-organization, Ooijpolder-Groesbeek

## Abstract

Biodiversity restoration on a landscape level requires people with different backgrounds to connect and collaborate over an extended period of time. Hence, understanding how conservation and restoration goals are negotiated and achieved necessitates an understanding of the dynamics of the social fabric: the social networks and interactions that develop, underpin, and sustain collective action. This paper identifies patterns and factors that have contributed to constructive collaboration for biodiversity in the rural area of *Ooijpolder-Groesbeek*, which has been at the vanguard of nature and landscape development in the Netherlands. We conducted a historical analysis of the period between 1985 and 2022, based on a broad range of literature and interviews with key actors in the region. We provide a narrative account of the tipping points and the preceding processes that propelled the region to its current state. The emergence of these tipping points is analyzed through the lens of a conceptual framework on the dynamic interplay between practices, social interactions, events, and circumstances. Our findings reveal how an integrative landscape approach, the use of suitable boundary objects, and continuous network building and relation management across various levels have contributed to the success of the collective effort.

## Introduction

Sustainable nature management and conservation have become increasingly important in the light of unprecedented biodiversity loss. Much attention is put on the role of land-use and, in particular, on agricultural practices as direct drivers of biodiversity loss (IPBES, 2019). Transforming those practices presents a multi-faceted challenge due to the intertwinement of biological and social processes, spanning multiple spatial and temporal scales (Fischer et al. 2021). As a result, rural areas in the Netherlands and elsewhere have become arenas of conflict and contestation in which a vast number of actors have to reconcile disparate interests with regard to housing, infrastructure, industry, agriculture, recreation, and nature (Aarts et al. 2007). At the same time, the pursuit of alternative pathways to socio-ecological issues is impeded by a high degree of complexity, continuous change, (knowledge) uncertainty, value conflicts, power struggles, and unintended consequences (Dentoni et al. 2018).

It is broadly acknowledged that collaboration across organizational, geographical, and juridical scales is key to halting and reversing the loss of biodiversity. Yet, much is left to learn about the circumstances under which collaboration for biodiversity recovery lives up to its promises. According to Cockburn et al. (2018; 2020) and others (Carpenter et al. 2012; Nkhata et al. 2008), there is an urgent need for contextualized, place-based research that pays attention to the temporal and social-relational dynamics as well as to the contextual processes that mediate and sustain collective action. Up until this point, only a few studies have addressed the long-term dynamics of social relationships in the context of social-ecological systems (SES) (e.g., Imperial et al. 2016; Kauneckis and Imperial, 2007; Ostrom, 1990).

With this study, we aim to deepen our understanding of how people in a local context mobilize other actors over time and across (organizational, professional, geographical) boundaries to trigger collective action for the recovery of biodiversity. We incorporate the proposal put forth by Winkelmann et al. (2022) and direct analytical attention to the processes and events leading up to tipping points in nature and land use management. While our primary focus is on the relational and temporal aspects of collaboration for biodiversity recovery, we heed Ostrom’s call for a configurational understanding of change processes (2007). Thus, we place special emphasis on examining the interplay of events, processes, and feedback loops that ultimately drive qualitative changes in the environment. Our primary focus centers on changes within the nature-agriculture nexus, given the significance of biodiversity recovery in this context.

## Theoretical Foundation

In recent years, an increasing number of scholars have started to utilize theories and concepts from the complexity sciences, in particular from the literature on SES, in order to better understand planned and unplanned changes in our environment. The notion of SES envisions humans and non-humans as part of interdependent and connected systems that are continuously changing and nested at different scale levels (Preiser et al. 2018).

Since the 1990’s, the cross-fertilization between natural and social sciences has led to the development of numerous state-and-transition models to understand the long-term dynamics of SES and the circumstances under which these systems may move from one state to another (Holling, 1973; Kingdon, 2011; Zahariadis, 2007). It is generally accepted that change in SES denies linearity and is practically unpredictable. It is believed that SES are complex, adaptive systems that evolve on the basis of the continuous interactions of their interdependent components. Yet, instances of serendipity, unintended consequences, and unforeseeable events, and feedback loops can have substantial influence on the trajectory of SES (Berkes, 2006).

A central concept commonly employed to understand change processes in SES is that of *tipping points*, referring to critical thresholds beyond which a system undergoes a rapid and possibly irreversible change. Reaching a tipping point typically involves a change in boundary conditions, either by weakening forces that maintain the initial state or by strengthening reinforcing positive feedbacks that amplify change. Hence, SES scholars are particularly interested in the phase preceding the tipping points and the interplay of variables that lead to their occurrence (Lenton et al. 2022). In recent years, the tipping point concept has been embraced by a variety of different disciplines to study an array of social phenomena, such as collective action, behavioral contagion, intractable conflicts, racial segregation, diffusion of innovation, or changes in policy domains and technology (See Lenton et al. 2022, for a comprehensive review on different conceptualizations and applications).

**Fig. 1:**
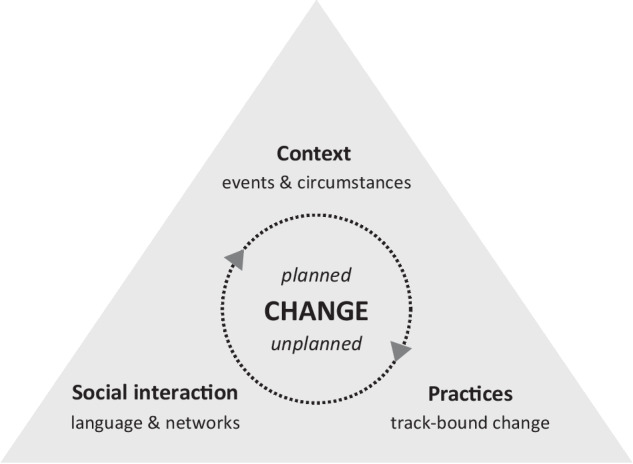
The interplay of change processes in social-ecological systems. Adapted from Van Woerkum et al. (2011)

The tipping point concept appears particularly valuable for studying historical developments in nature and land use management as it helps to identify critical moments of transformative change and pinpoint key events and interactions. Yet, as mentioned before, tipping points as such are only the result of the interplay between various interacting variables. Based on extensive empirical research in the context of spatial planning, environmental policy, and the management of public goods, Van Woerkum et al. (2011) found that changes within these domains are indeed the result of a continuous interplay of planned and unplanned processes. To make sense of real-life phenomena, they developed an analytical framework that distinguishes between three sources of change: (1) change driven by events and circumstances, (2) change driven by social interaction, and (3) change driven by practices (see Fig. 1).

In the following, we discuss these three dimensions and develop a better understanding of how these sources of change are relevant to our context of collaborative nature and land use management.

### Context Driven Change

This dimension refers to the confluence of coincidences and changing circumstances due to planned and unplanned events. Events, such as natural disasters, policy shifts, or technological advancements, can act as triggers and lead to rapid changes in SES. Moreover, changing circumstances, such as shifts in societal values, economic conditions, or ecological dynamics, continuously influence the course of collaborative efforts. Kingdon (1984, p. 206) for example, analyzed sudden changes in the policy domain and found that such changes are based on “considerable doses of messiness, accident, fortuitous coupling, and dumb luck”. Van Woerkum et al. (2011) further stress that also planned events are likely to have unintended consequences or might fall together with other events, potentially causing new and unforeseeable developments.

### Practice Driven Change

Practices are understood as patterns of behavior and activity that follow a certain script (execution of a plan) or that are repeated over time and eventually become habitual (routines and habits). At first glance, habits and routines suggest stability and structure rather than change. Van Woerkum et al. (2011) point out that it is indeed those routinized patterns of behavior that become entrenched over time and that can be resistant to change (path-dependency). For example, certain farming practices such as plowing, monoculture cropping, the use of chemical fertilizers and pesticides, and intensive land use have gradually become established and are nowadays constituting mainstream agriculture.

Just as our interactions with other people are never quite the same, so are our practices subject to and a source for change as they are performed and encountered in different contexts. Practices can change or be replaced due to new rules, social norms, technology, or infrastructure. An obvious source of change are the practices that are directly linked to the execution of a plan or policy. However, in many instances, the link between planned change and actual changes in practice is much less obvious and often subject to unintended consequences. Besides these ‘external stimuli’ to change one’s practices, Van Woerkum et al. (2011, p. 151) recognize the intrinsic potential of practices as a source of change. They argue that “in doing things, we experience impulses that we could not experience without action”, thereby drawing attention to the transformative potential of bodily experience as a source of change (learning by doing). It is hence important to note that practices are, just as social interactions, dualistic in nature: they are shaped by and, in turn, do shape larger cultural, technological, and social patterns (see also Bourdieu, Giddens and Schatzki). In short, “practices have their own dynamics” (Van Woerkum et al. 2011, p. 151) and can either constrain change or lead to novel practices.

### Change Driven by Social Interaction

Van Woerkum et al. (2011) emphasize that change in social systems often arises from every-day social interactions between actors. Social interactions refer to exchanges, communication, and relationships among individuals, groups, and organizations involved in a spatial planning process or a collaborative endeavor. These interactions can encompass various forms of engagement, such as deliberation, negotiation, collaboration, competition, and conflict in formal and informal settings.

They draw particular attention to the role of language and discourses as they shape the way in which individuals perceive and interpret their experiences and the world around them. By attaching meaning, value, or significance to the words we use, we create a ‘second order reality’ (Watzlawick 1990)—an interpretation rather than a presentation of a phenomenon. This human ability of sense-making and interpretation happens in interaction (Dewulf et al. 2009) and can in itself be a source of change as it gives room to creativity, spontaneity, and novel ideas. Through language, individuals can challenge existing norms, values, and beliefs and propose new ways of thinking. However, language can also be used to reinforce dominant practices and obscure or manipulate information, particularly in situations where power dynamics are at play (Ford et al. 2002). Everyday conversations are, in that sense, scenes of everyday politics. Given that concepts such as biodiversity restoration or nature conservation are inherently normative, they can be defined, understood, mobilized, and prioritized differently by different stakeholders. This process of interactional framing at the micro-level has been identified as a powerful mechanism of agenda setting in the environmental policy domain (Van der Stoep et al. 2017).

The framework developed by Van Woerkum and colleagues draws on principles of complexity sciences, making it particularly well-suited for our analysis. It acknowledges the inherent complexity and unpredictability of collective action settings yet provides a structured approach to identifying and interpreting the processes and events leading to critical changes in the context of nature and landscape developments. By combining the theory of tipping points and underlying sources of change, we are equipped to turn to our empirical case.

## Materials and Analysis

**Fig. 2:**
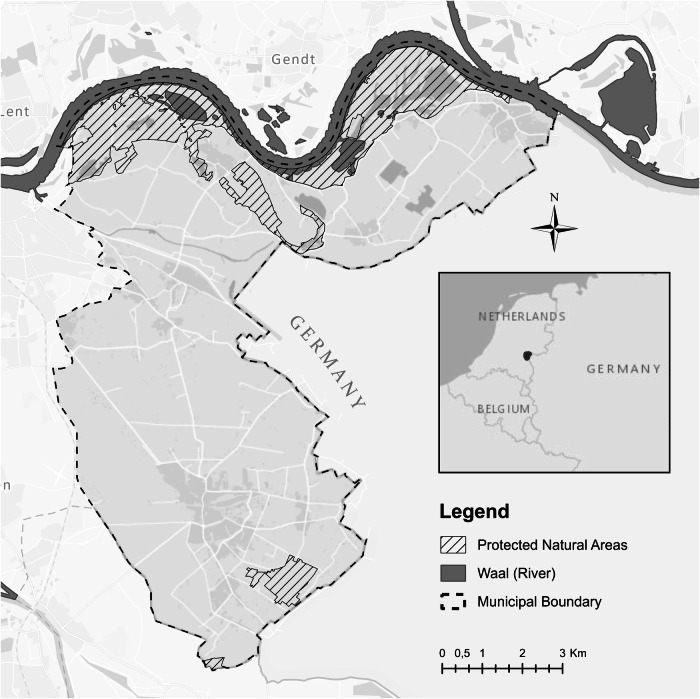
Map of the Ooijpolder-Groesbeek region. Geodata: Esri Nederland (2023) Community Map Contributors

The Ooijpolder-Groesbeek region (*OG*) is situated in the east of the Netherlands near the German border, with the river Waal to the north and the city of Nijmegen to the west, covering a land area of about 90 km^2^ and housing about 35,000 inhabitants (see Fig. 2). The region is nationally known as an exemplary case for nature and landscape development and conservation practices. Since the second half of the 20th century, the intensification of agriculture has started to threaten local flora and fauna. In the context of a broad land consolidation process in the 1980s, the increasing conflict over seemingly irreconcilable land use practices caused local actors to explore different pathways in order to halt the loss of biodiversity. Over the course of more than three decades, local farmers, conservationists, and other actors have joined forces to experiment with novel nature and landscape development and conservation practices.

Nowadays, large parts of the region are characterized by a diverse landscape with polders, floodplains, grass-, and farmland, intersected by landscape and water elements such as hedges, flower-rich dikes, pollard trees, and pools, forming a green mosaic of ecological connection zones and recreational hiking paths. Previous studies in this area indicate that biodiversity has improved over the past decades due to local restoration initiatives and a shift towards more collaborative forms of governance (Van Bussel et al. 2020). Ecological research throughout the years also confirmed an increase in landscape and species diversity in different parts of the area (Nijssen et al. 2014). Together, these studies suggest that local initiatives in this particular region contributed to demonstrable positive outcomes for landscape biodiversity. Our study aims to shed light on *how* local actors negotiated these objectives and how they managed to bring about and sustain novel practices in an ever-changing context.

The starting point for our explorative and qualitative study is the positive deviation of the case. To understand how changes with regard to nature and landscape development were negotiated and ultimately implemented, we employed a back-casting approach. While acknowledging the continuously changing nature of SES, we focused on tipping points, which signaled crucial moments in time and irrevocably influenced future developments. Adopting the analytical model by Van Woerkum et al. (2011) we analyzed patterns and dynamics leading up to those tipping points by investigating the interplay between events and circumstances, social interactions, and practices.

The data collection took place throughout various phases from December 2021 until June 2023. First, we built a historical profile of the study area based on secondary data, such as scientific publications, reports, minutes, policy documents, newsletters, and newspaper articles. The resulting timeline served as input for semi-structured interviews^1^. We conducted ten in-depth interviews with key informants who were involved in the region’s nature and landscape development over a long period of time. Participants were selected using snowball sampling, starting with a central, well-connected figure in the area. We intentionally included a diverse range of voices that reflected both the local community and the broader institutional context, including local and regional policy makers, conservationists, engaged farmers, and process facilitators.

The interviews were audio recorded and later transcribed. Analysis was performed using qualitative data analysis software (ATLAS.ti). We used coding in an iterative process, complementing primary data with grey literature and relevant scientific publications. We combined deductive coding using the concepts of the analytical model by Van Woerkum et al. (2011) with inductive coding, allowing for themes and links to emerge from the data. Categories were established through a process of thematization by identifying recurring tipping points and themes as well as the relations between those themes. While this approach allows for rich, qualitative insights, it also introduces the potential for researcher bias in data interpretation. To address this, we employed triangulation, using multiple data sources to validate findings, and engaged in peer discussions to challenge our interpretations, thereby striving to enhance the reliability of our analysis.

In the following, we begin by presenting the historical narrative that emerged from our analysis of interviews and historical documents. Here, we place an analytical focus on the interplay between context, practices, and social interactions within the study area. Subsequently, in the discussion section, we delve deeper into the patterns and dynamics that surfaced from our data. We interpret and discuss these findings, drawing on relevant literature to provide a robust theoretical grounding.

## Results

**Fig. 3:**
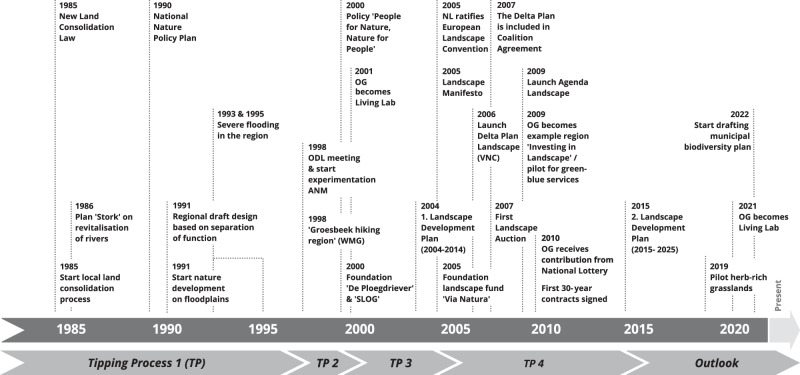
Timeline of tipping points and key events

The results are structured along a timeline (see Fig. 3) in which we distinguish four tipping points: at these critical junctures, an irreversible process is set in motion influencing future developments with regard to nature and landscape management in *OG*. We start each section by providing a brief description of the respective tipping point, followed by a detailed narrative account, and a concluding reflection on the interplay between changing circumstances, social interactions, and practices to interpret the processes that preceded those tipping points. The account is based on our data collection, as described in the previous section.

### Land Consolidation based on a ‘Separation of Function’

The land consolidation process set the stage for the (re)negotiation of land use practices in *OG*. Over the course of two decades (1985–2008), this process led to a strict spatial separation in the region: the outer dyke area (floodplains) of the river Waal and large parts of the Ooijpolder became dedicated natural areas for the purpose of nature development and restoration, whereas the inner dyke area was mainly dedicated to agricultural production. The underlying ‘separation of function’ greatly determined the future possibilities for nature and landscape conservation practices. Hence, it was referred to as a crucial tipping point by all respondents.

This development must be understood in relation to broader societal trends and changes in Dutch policy. In 1990, five years after the land consolidation process began, the Dutch government introduced its first *Nature Policy Plan*. With this plan, the government envisioned a connected network of ‘high-quality nature areas’ called the *‘National Ecological Network’* (LNV, 1990). Land consolidation was seen as a means to achieve this objective. The new policy signaled a break from traditional planning practices, which used to focus on conservation rather than the development of natural areas (Kuindersma et al. 2020).

This shift was sparked by a public debate on the value of human intervention in conservation efforts, with agriculture as a recurrent point of contention. While the traditional conservation discourse valued the cultural, man-made landscape with its interweaving of different functions, the new nature development discourse (also referred to as rewilding) rejected human interventions and instead imagined the restoration of largely unaffected, pre-human ecosystems (Verduijn et al. 2015; Van der Heijden, 2005).

The debate was also fought out locally and caused significant commotion among actors in *OG* as plans to renaturalize the Dutch rivers (including parts of the *OG* region) gained traction. Respondents recall heated discussions that reflected the two opposing viewpoints. The local plans in fact suggested a clear separation between cultivated (agricultural) land and natural areas (De Bruin et al. 1987).

Amidst the dispute, a local ecologist involved in the making of the plans and a supporter of the rewilding philosophy, joined the local land consolidation committee that was responsible for the drafting of the spatial rearrangement in the Ooijpolder. He later describes his involvement in the local land consolidation process as a means to bring this new vision for the river delta into practice (Arts et al. 2022). In 1991, the committee eventually presented a draft design for the spatial rearrangement of the area, indeed proposing a clear separation of function: less suitable agricultural plots on the floodplains were to be abandoned and agricultural activity were to be concentrated in the inner-dyke area, allowing for (further) intensification. The disputed mineral exploitation of sand and clay, which increasingly took place in the inner-dyke area, was eventually to be transferred to the floodplains (Smit, 1993). The profit from the extractions would offer the means to finance the start-up of one of the largest nature development projects in the Netherlands.

After the formal adoption of the land consolidation plan in 1996, the developments in the outer and inner dyke areas indeed proceeded along largely separate tracks. Separate in terms of both the practices that started to unfold as well as the alliances that were forged. After severe flooding in 1993 and 1995, the renaturalization plans for the floodplains gained considerable momentum as they were intended to improve water management in the region. Since then, the outer-dyke area has turned into an internationally acclaimed model for nature development (see e.g., Arts et al. 2022).

These developments illustrate how a changed context generated *social interactions* at various levels and moments, including public discourse on conservation practices and the renegotiation among local stakeholders. From these interactions *new practices* emerged, specifically the adjustment of land use strategies based on a separation of function rationale. This convergence of factors culminated in a substantial change in the physical composition of the landscape (spatial separation) and in shifting practices with regard to nature development and agriculture (rewilding in outer-dyke area and intensified agricultural practices in inner-dyke area). As we are particularly interested in the nexus between agriculture and conservation practices, the remainder of this chapter focuses primarily on the developments in the inner-dyke area, which has undergone a different process.

### Agricultural Nature Management: the Introduction of a New Practice

Thirteen years after the official start of the land consolidation process, a group of local farmers and nature conservationists started to experiment with a new practice: agricultural nature management. This practice significantly challenged the chosen path and introduced farmers as *green service providers*. After a period of experimentation, the group founded an agricultural member association called *de Ploegdriever* to formally enable the execution and contracting of agricultural nature management practices in the area. Simultaneously, a sister foundation (*SLOG*) was set up to acquire and manage small-scale nature plots and landscape elements. In the following, we describe the coming about of this new practice and its institutionalization.

As agriculture in the inner-dyke area intensified over the course of the land consolidation process, small-scale and mixed farming decreased due to competition, high investment costs, and tougher regulations. Dairy farming became more popular, and large fields of monoculture pasture began to appear on the landscape. The parcels of remaining farms increased in size, and previously uncultivated marginal land and edge strips were taken into production. Despite the land consolidation committee’s efforts to mitigate negative effects, many natural elements in the inner-dyke area had to give way to agricultural production and industrial infrastructure, affecting local fauna and flora. A local nature conservationist who closely monitored the land consolidation process recalls:

Many farmers eventually learned – there were, of course, rumors—that if you bring in bad plots with hedges and pollard trees [in the context of the land consolidation process], you’ll also get that kind of ‘handicapped’ plots back. So, you have to take all that out and level it out. Make sure it looks pretty. So, when the appraiser comes… Hence many farmers immediately started to dig up and destroy everything.

The spatial separation between agricultural production in the inner-dyke area and nature development practices on the floodplains was accompanied by a ‘social separation’ in terms of roles and responsibilities. A local farmer and chairman of the local farmers’ union started to question the division of labor and the contributions that the agricultural sector had to offer in relation to nature conservation. Coincidentally, he got in contact with a local biologist who was also the chairman of the environmental working group in Nijmegen. Together, they engaged in a dialog on the relationship between agriculture and nature and shared the ambition to bridge the growing divide in the region. This initial encounter evolved into a lasting friendship and a close collaboration that continues to this day, with many respondents referring to this partnership as an essential cornerstone for future success.

In 1998, an opportunity emerged to share their ideas with a broader audience. The *Foundation for Sustainable Agriculture* (ODL) invited representatives of farmers’ union and nature organizations to jointly explore possibilities for farmers to get involved in landscape and nature management in the region. Under the wings of ODL, the pair acquired the mandate and the financial means to experiment with this new kind of green service provision. Over the next two years, a dedicated group of farmers, backed by local conservationists, carried out various projects on behalf of municipalities, the forestry commission, the water board, and private landowners.

These projects primarily concentrated on managing and enhancing the cultural landscape, such as maintaining flowery field margins, hedges, and trees. They did not (yet) include management practices on the farm level. One of the initiators who first came up with the idea in *OG* explains:

The main idea [was] that the nature of the cultural landscape had to be improved. And the second [thought] was that farmers could play an important role in this regard while generating part of their income from it.

Having demonstrated that the practice was economically feasible and ecologically valuable, the group started to look for suitable organizational forms. A confluence of circumstances connected the group to the *Environmental Working Group Groesbeek* (*WMG*) – the local counterpart to the association in Nijmegen. The group had previously been in the spotlight for their campaign to preserve and restore a network of unpaved paths that was threatened by the land consolidation process. Rather than blaming farmers for the developments, the *WMG* was looking for a win-win for all parties involved. The model of agricultural nature management presented a unique opportunity that integrated ecological expertise with practical competencies. Finally, in 2000, the two-tier organization – *de Ploegdriever* and *SLOG* - was founded. According to one of the initiators, having the representatives of the local farmers’ union and the local environmental group on board was particularly crucial.

The great thing was that we connected with a number of key players, namely the chairmen of stakeholder organizations […]. There was a small group of frontrunners who embarked on this endeavor.

Reflecting on these developments reveals the profound impact of the *land consolidation plans* on *agricultural practices* (stimulating intensification in the inner dike area, removing landscape elements to increase efficiency) and *nature conservation practices* (intensifying rewilding efforts in the floodplains). These changes, in turn, reshaped *social interactions* as actors faced new realities, sparking once more a re-evaluation and renegotiation of *land use practices*. The establishment of *de Ploegdriever* and *SLOG* was instrumental in structuring collaboration between farmers and conservationists *(social interaction)*, and represented an important first step towards institutionalizing the *practice of agricultural nature management* in the region. This institutionalization, a key *change in context*, proved essential for the effective implementation and sustained maintenance of the regional green-blue network, as we’ll describe in the next section.

### The Landscape Concept: Towards an Integrative Regional Vision

The formulation of the first area-wide and integrative *Landscape Development Plan (LDP)* in 2004 constitutes the next tipping point in the development of the region. The plan outlined the vision for ‘nature and landscape’ in *OG* for a ten-year period and embedded the new practice of agricultural nature management in municipal policy. One of the objectives was the creation of an accessible and multifunctional *green-blue network*, connecting terrestrial (green) and aquatic (blue) elements of the cultural landscape, such as nature-friendly riverbanks, walking trails, flower-rich grasslands, and hedgerows. The provision of ‘green services’ by farmers and other landowners was an essential backbone for its implementation.

The introduction of agricultural nature management practice coincided with a shift in the broader policy landscape. In 2000, a new national nature policy was launched called *Nature for People, People for Nature* (LNV, 2000), which emphasized the multifunctionality of landscapes and promoted people’s participation in nature-related activities. Rural areas were to receive a ‘quality boost’ in terms of landscape, ecological, hydrological, and recreational aspects through the creation of *green-blue networks*. Given their significant landholdings, farmers were assigned a pivotal role in facilitating the successful implementation of these networks.

To experiment with and explore the financial, legal and practical aspects of this new approach, eight Living Labs were assigned across the Netherlands. Recognizing this unique opportunity, *de Ploegdriever* joined forces with two like-minded organizations to present *OG* as a suitable candidate region. One of these organizations was the *Association for Dutch Cultural Landscapes (VNC)*. Key to their proposal was a fair and sustainable compensation for farmers’ green service provision, which should compete on equal terms with conventional agricultural products. Respondents attribute the successful application to previous experience in agricultural nature management and the national reputation of the consortium partners, in particular the *VNC*.

The funding allowed for the establishment of a collaboration infrastructure, facilitating frequent coordination and social interaction between local actors. From this collaboration emerged the shared ambition to develop a long-term regional strategy for ‘nature and landscape’ in *OG*. Taking the historical identity and diversity of the landscape as a starting point, the Living Lab consortium set up a participatory process to evaluate and discuss ecological, cultural-historical, social-economic, and aesthetic aspects of the region. This process resulted in the first *Landscape Development Plan* (LDP) which, among others, envisioned a *green-blue network* in the inner-dyke area comprising 500 km of linear landscape elements.

According to respondents, structural cooperation from municipalities and the local farmers’ union was essential during the negotiation process, and networking and lobbying strategies were commonly employed. Some people in particular were perceived as indispensable for constructive collaboration and implementation in later years. The executive of the Province of Gelderland, for instance, recalls her motivation to recruit an acquaintance as project leader due to her boundary-spanning abilities and personal commitment. Together with the local biologist mentioned earlier, who has been a driving force behind the initiation of agricultural nature development in *OG*, they acted as the local task force, and they still continue to do so in various capacities to this day.

The *LDP* was complemented by an implementation program that translated the vision into more than 70 concrete projects. In order to communicate their vision, the committee developed a *quartets game*, illustrating possible combinations of landscape elements. Respondents note that this visual guide was crucial in conveying ideas across stakeholder groups. It also served as an entry point for conversations with individual farmers.

To ensure long-term financing and market-based incentives for the provision of green and blue services, the living lab consortium commissioned the establishment of a landscape fund (*Via Natura)*. However, the implementation of these plans faced considerable obstacles due to EU State Aid regulations, which viewed the compensation of green service provision through *public* means as potential market distortion. Over the next years, actors from *OG* extensively mobilized their connections to legal experts, provinces, ministries, and members of Parliament to lobby for their cause and gain leeway in the existing regulations (See Zwaan and Goverde, 2010, for a detailed analysis of the complex and lengthy process taking place between local, regional, national, and EU-level actors). The *OG* consortium ultimately lost the case, and only two projects under the ‘*green-blue network*’ track could be realized during the duration of the living lab project.

These developments illustrate how actors in *OG* created an enabling context for the implementation of their new vision, while building on a robust collaborative network (*social interaction*) and anticipating policy changes (*contextual change*). These changes were underpinned by a series of interrelated developments: the introduction of agricultural nature management in line with broader policy shifts towards multifunctional landscapes; the participatory formulation of the *Landscape Development Plan (LDP)* emphasizing green and blue service provision; and the resilience of local actors in overcoming setbacks to maintain momentum towards the implementation of a green-blue network. Despite obstacles, the established network and the strategic formulation of the *LDP* emerged as crucial factors in effectively capitalizing on emerging opportunities, setting the stage for further developments as discussed in the subsequent section.

### Long-term Perspective for Farmers: Creating Legal and Financial Conditions for Green Service Provision

While many actors focused on the realization of the *LDP* objectives within the given structures, others sought ways to challenge those structures. Key figures from *OG* called for action to halt the decline of biodiversity and proposed a plan for the conservation of cultural landscapes on national and European levels. Over the course of many years, the group garnered political and public support and developed a viable economic model to support its plans. Their proposal ultimately led to the establishment of four pilot projects, with *OG* becoming one of them. After a substantial contribution from the *National Postal Code Lottery*, the plans finally began to take shape. From 2010 to 2014, the *green-blue network* of landscape elements was realized on a larger scale, and its maintenance was ensured via *30-year contracts* with farmers. These contracts were considered the most significant turning point for nature and landscape management in *OG* as they guaranteed the development and maintenance of the green-blue network by providing a long-term financial perspective for farmers and other landowners.

One of the initiators of the previous living lab, the *Association for Dutch Cultural Landscapes (VNC)*, and in particular its director, had a national reputation for initiating legal procedures and challenging national policy in order to protect Dutch flora and fauna. Based in the heart of the region, the *VNC* sought to halt the loss of biodiversity and demonstrate an alternative path in its local environment. In 2006, the *VNC* launched the *‘Delta Plan for the Landscape’*, calling for a ‘quality boost for Dutch landscapes’ through the construction and restoration of 200,000 km of landscape elements along agricultural field margins (VNC, 2006). According to the *VNC*, caring for landscapes necessitates a market-based, sustainable payment system that is managed independently from the sway of shifting political preferences and short-term issues. Supported by corresponding publications and campaigns, the *VNC* lobbied extensively to garner support for their cause. To win the trust and cooperation of influential policy and political actors, the *VNC* made use of their reputation and extensive network connections. Both the director and the former adjunct director of the *VNC* referred to a particular strategy they employed in their quest to put their plan on the political and policy agenda. The adjunct director recalls:

All influential administrators and policymakers who have meant something to [the developments in] Ooijpolder-Groesbeek joined us in a search for badgers [at a particular location in OG]. We always talked about, ‘How are we going to turn the Delta Plan Landscape into a success?’

The Delta Plan has been endorsed by the *Landscape Manifesto*, a consortium of more than 40 national organizations representing nature conservation, landscape development, agriculture, and spatial planning. The consortium was established in late 2005 after the Netherlands ratified the European Landscape Convention. A respondent closely involved in the lobbying activities of the *VNC* explains the significance of the alliance’s support:

The alliance of the Landscape Manifesto has been very important for fostering public support. Because we could say that this is not only our plan; it’s the plan of 43 organizations, and hundreds of thousands of people are backing it. So, we are also relevant from an economic point of view. That is how we approached all those MPs and political parties.

The lobbying activities paid off, and the plan became part of the government coalition agreement in 2007, accelerating its implementation. Due to the plan’s novelty and financial uncertainty, a cost-benefit analysis was commissioned, and four pilot regions were chosen to test the ideas in practice. Since the *Landscape Development Plan* in *OG* was the result of an intensive exchange between *VNC* and local stakeholders, it contained considerable parallels with the *Delta Plan*. Previous efforts by local actors had demonstrated the feasibility, turning *OG* into an ideal candidate region. Despite initial hesitation due to the previous setbacks, the *VNC* managed to convince several local key figures to seize the opportunity, resulting in a successful application in 2009. The former project leader of the Living Lab, who also played a crucial role as coordinator of the landscape fund *Via Natura*, reflects on the reasoning behind seizing this opportunity:

We already knew how we wanted to do it here. We had all parties in place. […] It was, of course, VNC’s great achievement, that is undeniable. They are a national organization and have raised the issue politically.

The objective was the creation of an interconnected network of at least 500 hectares of landscape elements in *OG* that would allow for recreational activities. In order to proceed, a mix of financial streams from public and private sources needed to be secured. The latter was particularly important to avoid EU state aid regulations and to be able to offer farmers market-based prices. The VNC used their beneficiary status to apply for a one-time contribution from the *National Postal Code Lottery* and, with luck, was attributed 1.6 million euros. To manage the *private* money, a *Landscape Capital Foundation* was established, which still operates to this day. To secure and administer *public* means locally and in a sustainable manner, the *VNC* and other local actors continued to lobby across the board and leverage connections to local and regional governmental bodies built in previous years.

At the start of the project in 2009, municipalities, farmers’ union, water board, VNC, and private businesses formed a new committee to develop the legal and financial structures for the plan’s local implementation. Under the umbrella of the independent landscape fund *Via Natura*, the trusted duo that already played a pivotal role in previous years once again coordinated local efforts and facilitated the collaboration between representatives from public bodies, *SLOG, VNC*, the *WMG, de Ploegdriever*, and other local organizations. The consortium advanced quickly by leveraging long-term relationships in the field, previous experiences, and the network’s diverse expertize.

In 2010, pivotal progress was made as the first set of long-term contracts with landowners was signed, encompassing 500 contiguous hectares of landscape elements. A significant factor in farmers’ active engagement was the introduction of ‘landscaping business plans’ that allowed them to tailor the green service provision to the particularities of their farm and individual management practices. To ensure ecologically beneficial management, the contracts were linked to monitoring and control criteria, ensuring that the practices implemented aligned with environmental objectives. After achieving the initial objective, the focus area was expanded to include the southern part of the region (Groesbeek). Within a period of four years, a network of more than 45 km of landscape elements with recreational access was created, which is being maintained by local landowners to this day.

The signing of the 30-year contracts with farmers marked the pinnacle of the large-scale landscape development process in the inner-dyke area and the end of the pilot project in 2015. Yet, despite financial limitations preventing further scaling up, the involved actors displayed a remarkable dedication to enhancing their environment and pursuing the objectives outlined in the *LDP* in the subsequent years.

The narrative above underscores the pivotal role of systemic transformations *(changing context)* in fostering nature-inclusive practices at the grassroots level. At the same time, it is a testament to the potential influence of bottom up movements and social networks *(social interaction)* in driving transformative change. Actors in *OG* diligently sought to embed their integrative approach to nature and landscape conservation into formal structures and governance mechanisms, both on the national and local level. This alignment of local practices with institutional frameworks increased their legitimacy within the local community and among policymakers, facilitating the attraction of sustained investment and resources. The establishment of long-term contracts with farmers laid a solid foundation, ensuring the continuity and sustainability of *conservation practices* over time.

### Towards a Renewed Vision: New Networks and Shifting Discourses

Amid the current national turmoil surrounding issues like the nitrogen crisis, climate adaptation, and land use disputes, local actors in *OG* continue to seek collaborative ways to protect and enhance their environment. Notably, many of the actors who played a pivotal role during the past three decades are still actively engaged in regional development processes, and collaborative relationships are largely being sustained across the various organizations—a testament to the network’s resilience.

In addition, various citizen-farmer initiatives emerged in recent years, with each aiming to contribute to the improvement of biodiversity in their own respect: from community-based agriculture, short supply chain networks, agroforestry, environmental education, and joint nature restoration initiatives to small-scale experimentation with novel practices on the farm level. To maintain effective coordination between the various organizations and volunteer groups, a landscape community was set up, which was later replaced by a quarterly ‘partner meeting’ chaired by the municipality.

To ensure the maintenance of the green-blue network and to integrate new initiatives within municipal policy, a revised *Landscape Development Plan (LDP)* was formulated for the period between 2015 and 2025. Building on the vision established in 2004, the renewed *LDP* retained the landscape concept as a comprehensive framework to integrate ecological, socio-economic, cultural-historical, and aesthetic spatial planning aspects. In addition, more emphasis is being placed on monitoring activities to evaluate the ecological effects of certain practices and legitimize previous interventions.

As of July 2023, the drafting of a new municipal biodiversity plan is underway as a building block for a regional environmental vision (*omgevingsvisie*). This process has encouraged the formation of new alliances among individuals from nature and landscape organizations and citizen initiatives, advocating for the intrinsic and ecological value of nature and landscape in *OG*. As for provincial and national policy, recent efforts seem to have shifted towards encouraging *nature-inclusive practices* at the farm level to improve soil health and promote *agrobiodiversity*.

In 2021, *OG* received renewed impetus as it was designated once again as a living lab, now as part of a national research program focused on biodiversity restoration in rural areas. The present study has been conducted as part of the project and will be complemented by interdisciplinary research examining social-economic, legal-financial, and ecological facets of biodiversity restoration. Being transdisciplinary in nature, this project represents yet another building block in the history of nature and landscape conservation in *OG* and builds on the longstanding collaborations among local actors, such as *de* *Ploegdriever, SLOG, VNC, Via Natura*, the municipality, and others.

## Discussion

Our analysis exposes the complex interplay of various factors driving changes over time and shaping biodiversity restoration dynamics in the region. The case study illustrates how farmers, conservationists, local governments and other actors have gradually developed a collaborative and integrative approach to nature and landscape management. Central to this collaboration is the recognition of mutual dependencies —not just among the actors involved but also between local practices and wider societal changes. The synthesis table below (see Table 1) summarizes the key observations, providing a structured overview of the main changes observed within the study area and their link with our analytical framework.

**Table 1:** Synthesis of the Analytical Framework and the Main Findings from the Case

Analytical dimension	Concept	Findings from the case study
*Context driven change*	Changes driven by external events, shifts in societal values, policy changes, or technological advancements	▪ National debates and policy shifts towards nature development and conservation (e.g., Nature Policy Plan) ▪ Visible impact of agricultural intensification on rural landscapes ▪ Institutionalization of nature-inclusive practices on regional and national level ▪ Socio-ecological crises (e.g., flooding)
*Practice driven change*	Changes driven by alterations in routine activities, habits, and the execution of scripts and plans	▪ Agricultural nature management integrating ecological expertise with individual farming practices ▪ Development and implementation of local Landscape Development Plans (LDP) to scale up nature-inclusive practices ▪ Continuous experimentation, monitoring and adaptation of farming practices and their effects on ecological sustainability
*Change from Social Interaction*	Changes due to everyday social exchanges, the use of language, and networking	▪ Re-evaluation of local practices due to conflicting interests and perspectives with regard to land use practices and the ideal type of nature ▪ Engagement in catalytic conversations and integrative negotiations across professional boundaries leading to creative solutions, reframing of roles and responsibilities, and shared vision ▪ Establishment of regional organizations facilitating structured collaboration between different stakeholders ▪ Use of boundary objects and collective action frames to foster mutual understanding and shared goals. ▪ Involvement of boundary spanners and gatekeepers in fostering cross-boundary collaboration and leveraging personal access to networks and resources ▪ Maintaining long term commitment through clear task division, long-term contracts, continuous experimentation, small-scale projects and the celebration of incremental successes

In the following, we will discuss the main patterns by drawing on insights from complexity science, social movement theory, and communication science. Given our specific focus on the social-relational aspects and dynamics of multi-stakeholder collaborations, special attention is dedicated to the dimension of *social interactions*. We therefore take a closer look at the key factors and strategies that shaped the establishment and sustenance of actor relationships in *OG*.

### Integrative Negotiations

In the case of *OG*, the allocation of land for either nature conservation or agricultural purposes appeared as a win-win at first glance, allowing for the continuation and intensification of agricultural practices while ‘leaving space’ for nature to thrive. However, in retrospect, this approach disregarded potential synergies and feedback loops between agricultural practices and natural processes, resulting in (unintended) negative consequences for flora and fauna as well as in social conflicts.

From a negotiation theory perspective, the ‘separation of function’ can be seen as the start of a distributive negotiation between farmers and nature conservationists. Distributive negotiations are typically characterized by a win-lose mentality, where stakeholders compete for a limited set of resources or benefits. This often involves making concessions and trade-offs to reach an agreement that benefits both parties but may not result in an optimal outcome (Thompson et al. 2010). In this case, the separation of functions introduced an ‘either-or’ rationale, or, in other words, a zero-sum game. Such an approach can lead to adversarial relationships between stakeholders and hinder future collaboration (Innes and Booher, 2018; Pruitt and Carnevale, 1993). Indeed, previous research has shown that the introduction of the *Nature Policy Plan* led to tensions and conflicts with farmers across the Netherlands, who felt that their livelihoods were being negatively impacted by land use restrictions and the reduction of agricultural land for the sake of nature development (see e.g., Aarts and van Woerkum, 1995).

In *OG*, this conflict-ridden context appears to have sparked the need for collective action and a re-definition of the relationship between nature and agriculture on a local scale. The informal encounter between a local farmer and a local nature conservationist (see “Agricultural nature management: the introduction of a new practice”) exemplifies Baker’s notion of “catalytic conversations” (2010). According to Baker such spontaneous conversations characterized by empathic listening and joint fact-finding play a vital role in fostering innovation. They can carry significant transformative potential when occurring between people who act as connectors in the broader network, connecting people, narratives, resources, events, and plans (Van der Stoep et al. 2017).

Our case illustrates how small-scale social interactions evolved into a broader ‘counter-movement’, opting for a more integral approach to tackle the loss of biodiversity on agricultural land. According to the literature, an integrative negotiation approach requires actors to identify shared interests and interdependencies among each other and the issue at stake. The goal is to ‘create value’ for all parties involved by creating new options and finding creative solutions that meet the interests of all sides (Thompson et al. 2010; Lewicki et al. 2015). Ideally, this process is characterized by a high level of openness, a willingness to unravel underlying needs, values, norms, and objectives, and concern for ‘the other’ (Pruitt and Carnevale, 1993). Achieving this necessitates a shift from a narrow focus on individual gains to a broader perspective that encompasses the social and ecological dimensions of landscape and nature management. This process is known as *re-framing*, wherein actors collectively construct or redefine a common problem statement that, in this case, encompasses both ecological (biodiversity loss) and agricultural issues (existential & economic uncertainty) (Lewicki et al. 2015). Similarly, the literature on socio-ecological transformations refers to the *envisioning* of an alternative order, e.g., in terms of human-nature relationships, new management practices, and institutional structures (Moore et al. 2014; Olsson et al. 2004).

### Collective Action and the use of Boundary Objects

However, transitioning from a shared problem definition to collective action is not a straightforward process. Insights from social movement literature indicate that mobilizing actors around a new idea and initiating self-organization often requires the establishment of *collective action frames*. Such collective action frames serve as a means to render the complexity ‘out there’ intelligible, to define objectives, mobilize for action, identify relevant components (actors, species, processes) and guide human practices. They help to foster a sense of collective identity, solidarity, and commitment to the cause (Benford and Snow, 2000).

In the context of our study, collective action frames shape how stakeholders perceive and prioritize interdependencies between social and ecological processes. For instance, the framing of agricultural nature management emphasizes the shared goal of integrating nature-friendly practices within agricultural landscapes to protect and enhance biodiversity within the farmer’s context. It underlines the mutual benefits of sustainable farming practices for both agricultural productivity and ecological well-being while acknowledging the importance of preserving natural habitats and species diversity. Similarly, the ‘cultural landscape’ frame recognizes landscapes as products of human activity and emphasizes their cultural, historical, and ecological significance. This framing promotes integration, blurring the boundaries between nature conservation and agriculture, and can encourage sustainable landscape management practices. In more recent years, there has been a noticeable shift towards nature-inclusive agriculture, which focuses on management practices at the farm level.

This illustrates that collective action frames are not fixed but rather constructed, negotiated and sustained in continuous interaction (Dewulf et al. 2009). A common framework, such as the *Landscape Development Plan* and the related implementation program, can function as a tangible and shared reference point, allowing for the accommodation and bridging of the diverse perspectives and interests of actors. Such *boundary objects* (Star and Griesemer, 1989) indeed facilitated the interactional process of understanding ‘landscape and nature’ in *OG*, guided collective action and fostered a sense of ownership. Visualizations such as illustrations, maps, and quartet games served as mediators to facilitate communication, collaboration, and knowledge exchange between a diverse set of actors from different backgrounds.

### Fostering Relationships for Constructive, Cross-boundary Collaboration

The tightly woven network in *OG* facilitated collaboration, information sharing, and the cultivation of mutual respect and understanding. However, this network didn’t materialize overnight; instead, it evolved gradually over time, with several pivotal actors assuming central roles within the network. Among these key figures were gatekeeper personalities, who, alongside connectors, played a crucial role in shaping the flow of information and resources. These gatekeepers wielded significant influence, managing access to critical assets, networks and opportunities essential for advancing the collective cause. These key figures also possessed the skills to leverage personal and professional relationships across institutional and geographical scales in order to garner political and public support for the collective cause (also referred to as *boundary spanners*, see Aarts, 2018). Their credibility, trustworthiness, and sensitivity to network dynamics and the historical context were paramount in this process.

By opting for an integrative approach, local actors emphasized inclusion by creating an environment where individuals from diverse backgrounds could participate in the envisioning of alternative pathways. The integration of agricultural, economic, policy-related and ecological knowledge proved essential in fostering ecologically beneficial and socially viable practices. Local ecologists played a vital role as translators and representatives of the more-than-human perspective, contributing significantly to the sense-making process around socio-ecological interdependencies. By capitalizing on diversity in terms of knowledge, expertise and (soft) skills, actors could navigate different scales effectively, from local to national, and adapt swiftly to potential threats and emerging opportunities. This diversity was coupled with a clear task division based on actor’s individual strengths, leading to efficient teamwork and smart collaboration. This approach allowed actors to focus on tasks aligned with their expertise, promoting a sense of competence and empowerment within the *OG* actor network. The streamlining and coordination of strategic, administrative and practical tasks within the consortium and the broader network contributed to the success.

Last but not least, the determination and persistence of key actors played a pivotal role in ensuring the integration of nature-inclusive practices into local structures. Over time, their long-term commitment was sustained through continuous experimentation and the implementation of small-scale projects. Transition management research underscores the significance of such deliberate experimentation within “protected” spaces to explore the interdependencies and feedback loops in socio-ecological systems and to deal with uncertainty (Cumming et al. 2013; Geels, 2002). By closely monitoring the execution and the impact of the interventions, actors in *OG* could ensure that their collective efforts lead to ecologically beneficial outcomes. This iterative process not only facilitates social learning and adaptive management but also promotes interaction within the physical experimentation spaces. Simultaneously, they could build on incremental successes, which served as a positive feedback loop, reinforcing actors’ dedication to the collective effort. The visible impact of these successes played a vital role in gaining legitimacy and attracting the interest of local politicians and policymakers, as well as financial support.

## Implications for Practice

Based on our findings, we derive the following practical implications for practitioners involved in the design, facilitation, or governing of participatory multi-stakeholder processes for biodiversity restoration on a regional scale:

- *Historical awareness*: Actively seek knowledge of long- and short-term historical developments and previous agreements between actor groups. A (joint) ‘historical exploration’ can unravel interdependencies between the actors involved and help to recognize tensions to circumvent potential pitfalls in future interactions. The process as such can be useful in fostering mutual understanding of ‘how things have become’.
- *Working in established networks*: Consider working on a small scale and with established networks to enhance collaboration, trust, and efficiency in addressing complex socio-ecological issues and to leverage existing resources, knowledge, and relationships. Acknowledge grassroots movements and community-rooted individuals who naturally assume the role of connectors, adept at fostering communication and facilitating the exchange of knowledge. We also encourage practitioners to embrace the role of boundary spanners themselves by actively seeking new connections, reaching across networks, engaging with people who think and act differently and by building sustainable relationships.
- *Promoting inclusivity and diversity through dialogue*: Create an environment where individuals from diverse backgrounds can actively participate by facilitating open dialogue and adopting an integrative negotiation style, including joint fact-finding, the integration of different knowledge types, and concern for the actors involved. Investing in the creation of spaces and situations that encourage unscripted and informal conversations between actors holds the potential for sparking ‘catalytic conversations’.
- *Fostering continuous experimentation and learning*: Acknowledge the complex and uncertain nature of sustainability-related issues and the time it takes to achieve transformative change. Promoting an adaptive approach based on continuous experimentation, monitoring, evaluation, adjustment, and learning is crucial for exploring socio-ecological interdependencies. Celebrating and showcasing incremental and visible successes can help maintain motivation and support.
- *Translating complexity across actor groups*: Be aware of the value of boundary objects that are being deployed in the collaborative process. Invest in communication and translation across diverse actor groups, e.g., through visualization, kitchen table conversations, or first-hand experiential learning. Identify and support individuals who can serve as ‘translators’, particularly in contexts that require specialized knowledge from both ecological and social domains.

## Limitations to the Research

We conducted this study with great care and collected data until we reached theoretical saturation. That said, our study also has limitations. We acknowledge the subjective nature of historical analysis and recognize that our understanding of the past is shaped by both the evidence and by the contemporary perspectives of the researchers and participants. Despite efforts to critically evaluate and cross-reference historical documents and interview transcripts to mitigate bias, interpretations remain influenced by the present-day perspectives of those involved in the research. Furthermore, it is important to note that our study may not fully encompass the voices and contributions of individuals and groups who were excluded from or overlooked in the course of the historical developments under study.

The choice of the Ooijpolder-Groesbeek region as a case study for this research brings with it limitations in terms of generalizability. While this case provides valuable insights into the processes and challenges of fostering regional collaboration for biodiversity restoration, the findings may not be directly applicable to other contexts with different social, economic, and ecological conditions. Future research could benefit from comparing multiple case studies to explore the variability in collaborative efforts across different regions. We also repeat the call for contextualized and longitudinal studies to trace the evolution of collaborative networks and their adaptive strategies over extended timeframes. A promising avenue is the examination of boundary objects and collective action frames, shedding light on their role in fostering collaboration and knowledge integration within diverse stakeholder groups.

## Conclusion

Working on complex issues such as biodiversity restoration requires the involvement of actors from different backgrounds to delve into the intricate web of social-ecological interdependencies and to continuously negotiate and re-define the scale and scope of the collective effort. Our historical case study in the Dutch region of *OG* illustrates how local actors gradually developed a collaborative and integrative approach to nature and landscape management while aiming to bridge the growing divide between agriculture and nature conservation. By recognizing the value of diversity and capitalizing on relationships, actors in the region effectively leveraged their resources for ecologically beneficial outcomes. Moreover, the sensitivity demonstrated by the actors to the multilevel context enabled them to adapt swiftly to potential threats and seize emerging opportunities. The resilience displayed by the actors in *OG* can be attributed to their continuous efforts to build lasting relationships across boundaries. The integrative and collaborative approach adopted by the community has been a key factor in maintaining this resilience.

Our historical case study contributes to the literature on SES and environmental management by providing empirical evidence of effective strategies, successful collaborations, and the interplay of various factors in shaping sustainable nature and landscape management over time. We support the idea that building lasting relationships at the regional level and fostering social networks significantly enhances the ability to adapt to changing circumstances over time, foster better communication, and effectively seize windows of opportunity.

## Data Availability

The data is not accessible due to privacy restrictions.

## Footnotes

1 The interviews lasted between 60 and 106 min. Nine out of the 10 interviews were conducted face-to-face at a location of the respondent’s choice, one was conducted online due to time restrictions.
